# Effects of β-Adrenergic Blockade on Metabolic and Inflammatory Responses in a Rat Model of Ischemic Stroke

**DOI:** 10.3390/cells9061373

**Published:** 2020-06-01

**Authors:** Shih-Yi Lin, Ya-Yu Wang, Cheng-Yi Chang, Chih-Cheng Wu, Wen-Ying Chen, Yu-Hsiang Kuan, Su-Lan Liao, Chun-Jung Chen

**Affiliations:** 1 Center for Geriatrics and Gerontology, Taichung Veterans General Hospital, Taichung City 407, Taiwan; sylin@vghtc.gov.tw; 2 Institute of Clinical Medicine, National Yang Ming University, Taipei City 112, Taiwan; yywang@vghtc.gov.tw; 3 Department of Family Medicine, Taichung Veterans General Hospital, Taichung City 407, Taiwan; 4 Department of Surgery, Feng Yuan Hospital, Taichung City 420, Taiwan; c.y.chang.ns@gmail.com; 5 Department of Anesthesiology, Taichung Veterans General Hospital, Taichung City 407, Taiwan; chihcheng.wu@gmail.com; 6 Department of Financial Engineering, Providence University, Taichung City 433, Taiwan; 7 Department of Data Science and Big Data Analytics, Providence University, Taichung City 433, Taiwan; 8 Department of Veterinary Medicine, National Chung-Hsing University, Taichung City 402, Taiwan; wychen@dragon.nchu.edu.tw; 9 Department of Pharmacology, Chung Shan Medical University, Taichung City 402, Taiwan; kuanyh001@gmail.com; 10 Department of Medical Research, Taichung Veterans General Hospital, Taichung City 407, Taiwan; slliao@vghtc.gov.tw; 11 Department of Medical Laboratory Science and Biotechnology, China Medical University, Taichung City 404, Taiwan

**Keywords:** adrenergic system, microglia polarization, neuroinflammation, stroke

## Abstract

Ischemic stroke provokes an inflammatory response concurrent with both sympathetic nervous system activation and hyperglycemia. Currently, their crosstalk and consequences in stroke outcomes are of clinical attraction. We have provided experimental evidence showing the suppressive effects of the nonselective β-adrenoreceptor antagonist propranolol on hyperglycemia, inflammation, and brain injury in a rat model experiencing cerebral ischemia. Pretreatment with propranolol protected against postischemic brain infarction, edema, and apoptosis. The neuroprotection caused by propranolol was accompanied by a reduction in fasting glucose, fasting insulin, glucose tolerance impairment, plasma C-reactive protein, plasma free fatty acids, plasma corticosterone, brain oxidative stress, and brain inflammation. Pretreatment with insulin alleviated—while glucose augmented—postischemic brain injury and inflammation. Additionally, the impairment of insulin signaling in the gastrocnemius muscles was noted in rats with cerebral ischemia, with propranolol improving the impairment by reducing oxidative stress and tumor necrosis factor-α signaling. The anti-inflammatory effects of propranolol were further demonstrated in isoproterenol-stimulated BV2 and RAW264.7 cells through its ability to decrease cytokine production. Despite their potential benefits, stroke-associated hyperglycemia and inflammation are commonly linked with harmful consequences. Our findings provide new insight into the anti-inflammatory, neuroprotective, and hypoglycemic mechanisms of propranolol in combating neurodegenerative diseases, such as stroke.

## 1. Introduction

Stroke is a leading cause of adult long-term disability, mortality, and morbidity worldwide. Currently, thrombolytic therapy with tissue plasminogen activator (tPA) still remains the first option for the treatment of ischemic stroke, although only a small population of patients have seen any clinical benefits due to its narrow therapeutic window after stroke onset [1]. Additionally, the increased risk of intracerebral hemorrhagic transformation and its accompanied inflammation further exhaust the beneficial effects of tPA. Clinical findings indicate that hyperglycemia is critical in counterbalancing the therapeutic benefits and increasing the adverse complications of tPA through an action mode related to inflammation exacerbation [2,3,4,5]. Despite their protective and regenerative potential, overwhelming hyperglycemia and neuroinflammation have been implicated in the pathogenesis of stroke. Rodent studies have also revealed the crosstalk among tPA, hyperglycemia, and neuroinflammation in cerebral ischemic brain injury, and the existing therapeutic benefits by means of targeting hyperglycemia and neuroinflammation [6,7,8,9,10]. These phenomena underscore the importance of exploring the underlying mechanisms of stroke-accompanied hyperglycemia and neuroinflammation, as well as highlighting their therapeutic potential towards combating stroke disease and related complications.

There is existing bidirectional communication between the immune system and the central nervous system (CNS) [11]. Upon CNS injury, the activated immune system displays cytotoxic effects at the early phase, but removes debris and promotes tissue regeneration during the late phase. Oppositely, the case of immunosuppression increases the risk of infectious complications and worsens the outcome. Currently, the involvement of the sympathetic nervous system and hypothalamic–pituitary–adrenal (HPA) axis in stroke-accompanied immune suppression and immune activation has been described [12,13,14,15,16,17,18]. Because of the activation of sympathetic tone, and how the HPA axis is seen in acute ischemic stroke, as well as their dual roles in immune activities, the sympathetic nervous system and the HPA axis represent alternative targets for intervention, with an aim towards the treatment of stroke.

Amongst the distinct adrenoreceptor subtypes, β-adrenoreceptors attract much attention in the aspect of immunity. Activation of β2-adrenergic signaling increases postischemic brain infarction in mice [19]. On the contrary, β-adrenoreceptor antagonists provide neuroprotection against cerebral ischemic brain injury [20,21,22]. Both in vivo and in vitro, adrenaline/noradrenaline and β-adrenoreceptor agonists show an immunocompetent ability leading to neuroinflammation by targeting microglia [23,24,25,26]. Since microglia play a substantial role in stroke-accompanied neuroinflammation and neurodegeneration [27], β-adrenergic antagonists are theoretically candidates for the development of therapeutic options regarding stroke.

Previously, we had detected an elevated circulating level of adrenaline, noradrenaline, and corticosterone in a rat model of cerebral ischemia, and reported the improved effects on adipose inflammation, hepatic inflammation, and hepatic gluconeogenesis, which the nonselective β-adrenoreceptor antagonist propranolol had [14,15,16,28]. Luger et al. [22] further demonstrated that propranolol is able to ameliorate stroke-associated impairment of glucose tolerance and brain ceramide accumulation. Clinically, despite their variability, the use of β-adrenoreceptor antagonists is advisable for patients at risk of stroke [29]. To extend the scope of propranolol’s biological implications, its anti-inflammatory and neuroprotective identities were further explored in a rat model of cerebral ischemia.

## 2. Materials and Methods

### 2.1. Cerebral Ischemia and Treatments

**Figure 1:**
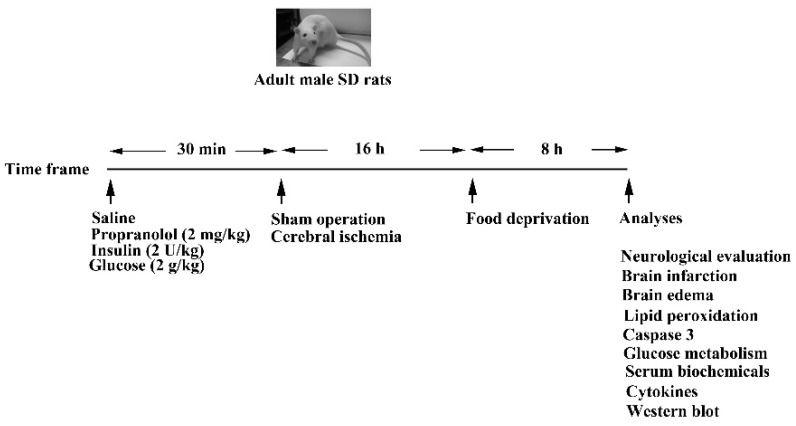
Schematic diagram of animal study design. Adult male Sprague-Dawley rats were intraperitoneally administrated with saline, propranolol (2 mg/kg), insulin (2 U/kg), or glucose (2 g/kg), 30 min prior to sham or cerebral ischemia for a course of 24 h. The last eight hours, rats were deprived of foods except drinking water. At the end of the experiments, rats were allocated into the indicated analyses.

The protocols surrounding animal study were reviewed and approved by the Animal Experimental Committee of Taichung Veterans General Hospital and strictly adhered to as per the Institute’s guidelines (La-1071584, 1 August 2018). Adult male Sprague-Dawley rats weighing 300–350 g (*n* = 108 in total, with the specific numbers of rats indicated at the corresponding experiments) were anesthetized with isoflurane (2–4%), and body temperatures were maintained at 37.0 ± 0.5 °C. Focal cerebral ischemia was produced by clamping the two common carotid arteries and the right middle cerebral artery, as described previously [14]. For the sham operation, all surgical procedures were performed except for arterial occlusion. A single bolus of normal saline, propranolol (2 mg/kg), insulin (2 U/kg), or glucose (2 g/kg) was intraperitoneally delivered to ischemia or sham rats 30 min prior to surgery. All the ischemia and sham rats receiving treatments were euthanized for analyses 24 h after completion of surgery. A schematic diagram of the animal study is shown in Figure 1.

### 2.2. Neurological Evaluation

A modified six-point neurological deficit severity scoring criteria was applied in order to evaluate sensorimotor performance, which was done by technicians who had been blind to the treatments (*n* = 6 per group) [27].

### 2.3. Quantification of Ischemic Infarction

Rats (*n* = 6 per group) were anesthetized with isoflurane (2–4%) and then decapitated. The dissected brains were put into a Brain Slicer Matrix and sliced into a serial coronal section at 2 mm intervals. The brain sections were then immersed in 2% triphenyltetrazolium chloride (TTC) solution at 37 °C for 30 min, followed by fixation in 10% phosphate-buffered formalin for 45 min [27]. The areas of brain infarction were highlighted by a white color and the volume was measured with a computer image analysis system (IS1000; Alpha Innotech Corporation, San Leandro, CA, USA).

### 2.4. Brain Edema

Rats (*n* = 6 per group) were anesthetized with isoflurane (2–4%) and then decapitated. The dissected brains were separated into contralateral and ipsilateral hemispheres for the isolation of cortical tissues. The obtained contralateral and ipsilateral cortical tissues were dried in an oven at 110 °C for 24 h. The water content was calculated by the wet/dry weight method, as described previously [27].

### 2.5. Measurement of Lipid Peroxidation

Rats (*n* = 6 per group) were anesthetized with isoflurane (2–4%) and then decapitated. The dissected brains were separated into contralateral and ipsilateral hemispheres for the isolation of cortical tissues. The obtained contralateral and ipsilateral cortical tissues and gastrocnemius tissues were subjected to the measurement of lipid peroxidation using a thiobarbituric acid-reactive substance (TBARS) assay kit (Abcam, Cambridge, UK). TBARS is expressed as malondialdehyde (MDA) equivalents.

### 2.6. Caspase 3 Activity Assay

Rats (*n* = 6 per group) were anesthetized with isoflurane (2–4%) and then decapitated. The dissected brains were separated into contralateral and ipsilateral hemispheres for the isolation of cortical tissues. The obtained contralateral and ipsilateral cortical tissues were subjected to the measurement of caspase 3 activity using a commercial fluorometric protease assay kit (BioVision, Mountain View, CA, USA).

### 2.7. Glucose Tolerance Test

Prior to an intraperitoneal glucose tolerance test (IPGTT) (*n* = 6 per group), rats were deprived of diet for 8 h. The IPGTT was performed through the intraperitoneal administration of glucose solution (2 g/kg body weight). Their blood was then collected from the tail veins over time and glucose levels were measured using a hand-held Accu-Check glucometer (Roche Diagnostics, Indianapolis, IN, USA). The total area under the curve (AUC) for IPGTT was calculated using the trapezoidal (trapezium) rule.

### 2.8. Blood Sample Analyses

Rats (*n* = 6 per group) were anesthetized with isoflurane (2–4%); their blood was withdrawn from the left femoral artery and the plasma samples kept at −80 °C until the analyses. The plasma levels of insulin (Shibayagi, Gunma, Japan), C-reactive protein (CRP), free fatty acids, and corticosterone (R&D Systems, Minneapolis, MN, USA) were measured using enzyme-linked immunosorbent assay (ELISA) kits, according to the manufacturer’s instructions.

### 2.9. Measurement of Tissue Cytokines

Rats (*n* = 6 per group) were anesthetized with isoflurane (2–4%) and then decapitated. The dissected brains were separated into contralateral and ipsilateral hemispheres for the isolation of cortical tissues. The obtained contralateral and ipsilateral cortical tissues and gastrocnemius tissues were subjected to the measurement of Tumor Necrosis Factor-α (TNF-α) protein content using ELISA kits (R&D Systems, Minneapolis, MN, USA).

### 2.10. Western Blot Analysis

Rats (*n* = 6 per group) were anesthetized with isoflurane (2–4%) and then decapitated. The dissected brains were separated into contralateral and ipsilateral hemispheres for the isolation of cortical tissues. The obtained contralateral and ipsilateral cortical tissues and gastrocnemius tissues were subjected to protein extraction using the commercial tissue protein extraction reagents (T-PER, Pierce Biotechnology, Rockford, IL, USA). The obtained proteins went through regular SDS-PAGE separation, electrical transfer, antibody reaction, and enhanced chemiluminescence Western blotting reagents. The visualized signals were quantitated using a computer image analysis system (IS1000; Alpha Innotech Corporation, San Leandro, CA, USA). Proteins recognized by the primary antibodies were: p38 (sc-7149, 1:1000), Akt (sc-377457, 1:1000), phospho-Akt (sc-7985, 1:1000), interferon regulatory factor 8 (IRF8, sc-365042, 1:1000), tumor necrosis factor-α receptor type I (TNFRI, sc-7895, 1:1000), glial fibrillary acidic protein (GFAP, sc-6170, 1:1000), zonula occludens-1 (ZO-1, sc-10804, 1:1000), erythroid 2-related factor-2 (Nrf2, sc-365949, 1:1000), Sirt1 (sc-15404, 1:1000), glyceraldehyde 3-phosphate dehydrogenase (GAPDH, sc-32233, 1:1000) (Santa Cruz Biotechnology, Santa Cruz, CA, USA), c-Jun N-terminal kinase (JNK, 1478-1, 1:1000) (Epitomics, Burlingame, CA, USA), phospho-p38 (612281, 1:1000), phospho-JNK (612541, 1:1000), cyclooxygenase 2 (COX-2, 610204, 1:1000), insulin receptor substrate-1 (IRS1, 611395, 1:1000) (BD Biosciences, Franklin Lakes, NJ, USA), Cluster of Differentiation 68 (CD68) (MCA341R, 1:1000), CD163 (MCA342R, 1:1000) (Bio-Rad Laboratories, Hercules, CA, USA), phospho-IRS1 (S307, 23381, 1:1000), and microtubule-associated protein-2 (8707, 1:1000) (Cell Signaling, Danvers, MA, USA).

### 2.11. Cell Cultures

The murine microglia BV2 cell line and macrophage RAW264.7 cell line were maintained in Dulbecco’s Modified Eagle Medium (DMEM) containing 10% fetal bovine serum (FBS) [30]. BV2 and RAW264.7 cells were pretreated with a vehicle or propranolol (10 μM) for 30 min before being incubated with isoproterenol (0 and 10 μM) for an additional 24 h. The cell cultured supernatants (100 μL) were subjected to the measurement of TNF-α and interleukin-6 (IL-6) using commercial ELISA kits (R&D Systems, Minneapolis, MN, USA). For nitric oxide (NO, nitrite/nitrate) determination, the samples were subjected to the measurement using a Griess reagent kit (Thermo Fisher Scientific, Waltham, MA, USA).

### 2.12. Statistical Analysis

All statistical results are presented as mean ± standard deviation. A one-way analysis of variance was performed in order to evaluate experimental values between groups, with a consequent Dunnett’s test or Tukey post-hoc test performed for the purpose of comparison. It was considered statistically significant when the *p* value was less than 0.05.

## 3. Results

### 3.1. Propranolol Alleviated Postischemic Brain Injury

**Figure 2:**
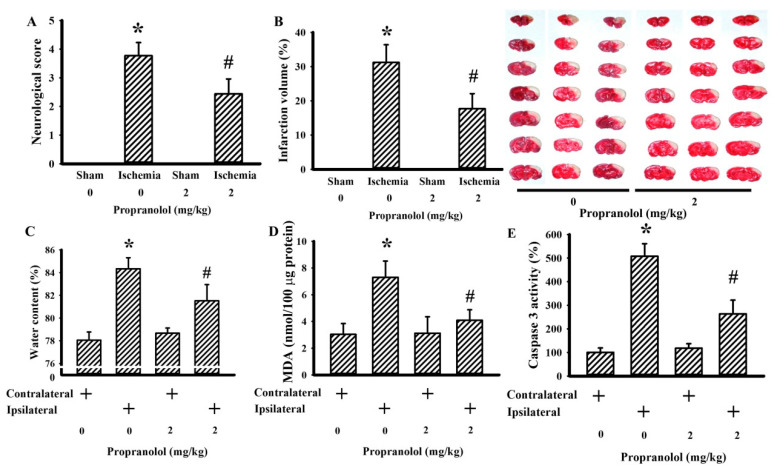
Propranolol protected against cerebral ischemia injury. Rats receiving a normal saline vehicle or a propranolol (2 mg/kg) intraperitoneal injection were subjected to sham or permanent cerebral ischemia for 24 h. (**A**) Neurological deficits were evaluated by neurological score. (**B**) Representative photographs show the histological examination of a brain infarction by triphenyltetrazolium chloride (TTC) staining. The average percentage of infarction volume in the ipsilateral hemisphere is depicted. (**C**) The water content in the contralateral and ipsilateral cortical tissues was measured. (**D**) The contents of malondialdehyde (MDA) in the contralateral and ipsilateral cortical tissues were measured. (**E**) Proteins were extracted from the contralateral and ipsilateral cortical tissues and subjected to an enzymatic assay of caspase 3 activity. * *p* < 0.05 vs. sham/saline or the contralateral tissues of the vehicle groups and # *p* < 0.05 vs. ischemia/saline or the ipsilateral tissues of the vehicle groups, *n* = 6.

To investigate the neuroprotective potential against cerebral ischemia brain injury, propranolol was delivered into rats 30 min prior to ischemia. Permanent cerebral ischemia caused neurological deficits (Figure 2A), brain infarction (Figure 2B), brain edema (Figure 2C), elevation of MDA (Figure 2D), and increased caspase 3 activity (Figure 2E). Propranolol alleviated the postischemic changes (Figure 2), implying that pretreatment with propranolol protects the brain against cerebral ischemia injury.

### 3.2. Propranolol Alleviated Postischemic Inflammation

**Figure 3:**
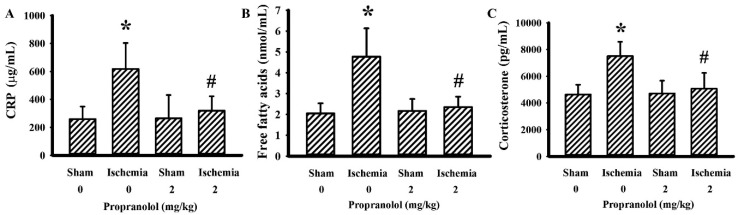
Propranolol alleviated postischemic changes in plasma biochemicals. Rats receiving a normal saline vehicle or a propranolol (2 mg/kg) intraperitoneal injection were subjected to sham or permanent cerebral ischemia for 24 h. The blood samples were collected and subjected to the measurement of C-reactive protein (CRP) (**A**), free fatty acids (**B**), and corticosterone (**C**). * *p* < 0.05 vs. sham/saline group and # *p* < 0.05 vs. ischemia/saline group, *n* = 6.

**Figure 4:**
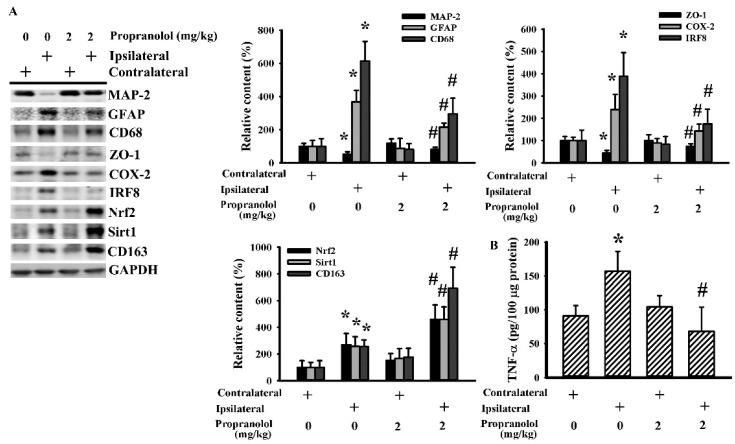
Propranolol alleviated postischemic brain inflammation. Rats receiving a normal saline vehicle or a propranolol (2 mg/kg) intraperitoneal injection were subjected to permanent cerebral ischemia for 24 h. Proteins were extracted from the contralateral and ipsilateral cortical tissues and subjected to Western blot (**A**) with the indicated antibodies or ELISA for the measurement of TNF-α (**B**). * *p* < 0.05 vs. the contralateral tissues of the vehicle group and # *p* < 0.05 vs. the ipsilateral tissues of the vehicle group, *n* = 6.

To explore the inflammatory changes, parameters of inflammation were determined in both blood samples and brain tissues. The circulating levels of CRP (Figure 3A), free fatty acids (Figure 3B), and corticosterone (Figure 3C) were elevated in rats with cerebral ischemia, with the increments being alleviated by propranolol. Elevated COX-2 (Figure 4A) and TNF-α (Figure 4B) protein levels were detected in ipsilateral cortical tissues after cerebral ischemia. Parallel elevation was noted in astrocyte-associated GFAP, macrophage/microglia lineage-associated CD68, and activated microglia-associated IRF8. On the contrary, the levels of neuron-specific Microtubule-Associated Protein 2 (MAP-2) and tight junction ZO-1 protein were downregulated by ischemia. Those changes in cerebral ischemia were reversed by propranolol (Figure 4A). Intriguingly, the expression of alternatively activated microglia-accompanied CD163, Nrf2, and Sirt1 was increased by propranolol (Figure 4A). These findings indicate that propranolol has a negative effect on cerebral ischemia-activated systemic and brain inflammation.

### 3.3. Propranolol Improved Postischemic Hyperglycemia

**Figure 5:**
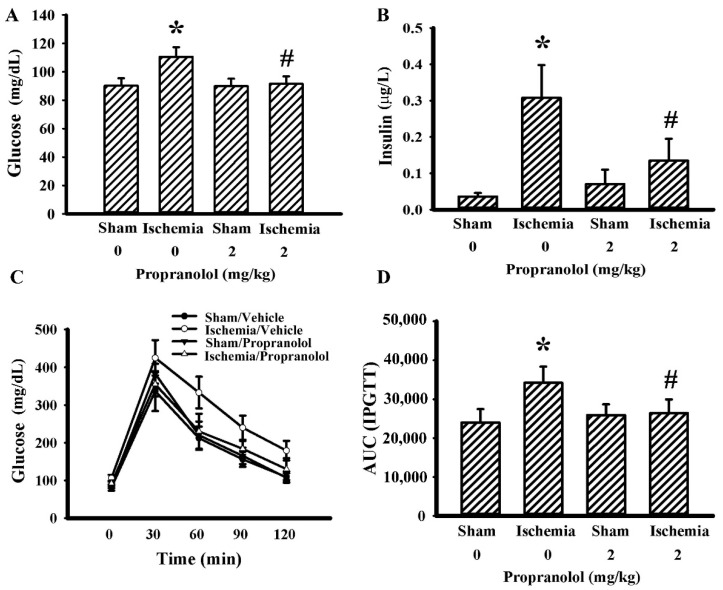
Propranolol alleviated postischemic hyperglycemia. Rats receiving a normal saline vehicle or a propranolol (2 mg/kg) intraperitoneal injection were subjected to sham and permanent cerebral ischemia for 24 h. The blood samples were collected from 8 h fasting rats and subjected to glucose (**A**) and insulin (**B**) measurement. The 8 h fasting rats were intraperitoneally injected with a glucose solution (2 g/kg). Blood samples were collected from the tail veins at the indicated times after treatments and the levels of glucose were measured (**C**). The area under the curve (AUC) of the glucose time curves was calculated (**D**). * *p* < 0.05 vs. sham/saline group and # *p* < 0.05 vs. ischemia/saline group, *n* = 6.

Parameters of glucose metabolism were determined in fasting rats. Cerebral ischemia brought hyperglycemia (Figure 5A) and hyperinsulinemia (Figure 5B) upon the rats, while a reverse effect has been observed in propranolol rats. Next, the effects of propranolol on postprandial glucose dynamics were evaluated. Postischemic rats had higher postload glucose levels after the intraperitoneal glucose injection, while the postload glucose levels were decreased by propranolol (Figure 5C,D). These findings suggest there is a beneficial effect surrounding propranolol against postischemic hyperglycemia, hyperinsulinemia, and impaired glucose tolerance.

### 3.4. Insulin and Glucose Had Opposite Effects on Postischemic Changes

**Figure 6:**
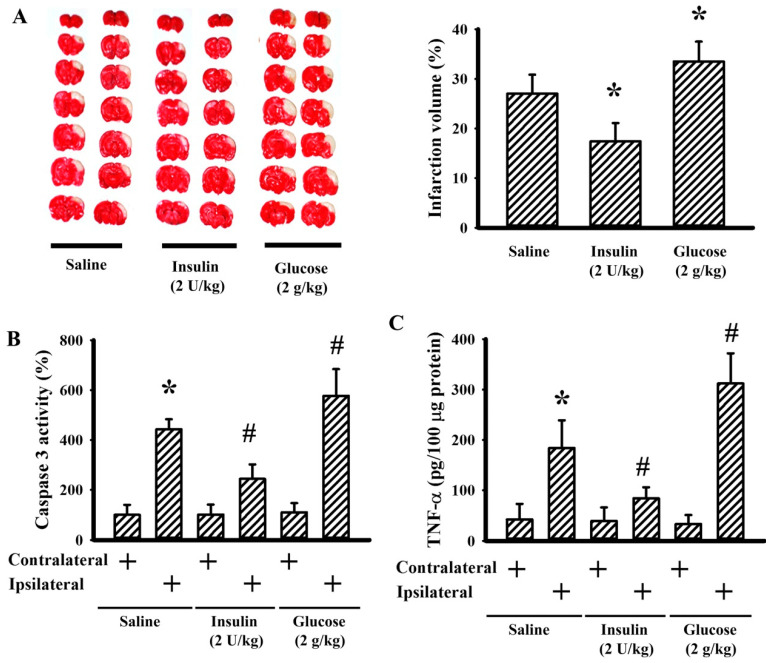
Insulin reduced but glucose augmented cerebral ischemia injury. Rats receiving a normal saline vehicle, insulin (2 U/kg), or a glucose (2 g/kg) intraperitoneal injection were subjected to permanent cerebral ischemia for 24 h. (**A**) Representative photographs show the histological examination of brain infarction by TTC staining. The average percentage of infarction volume in the ipsilateral hemisphere is depicted. (**B**) Proteins were extracted from the contralateral and ipsilateral cortical tissues and subjected to an enzymatic assay of caspase 3 activity. (**C**) Proteins were extracted from the contralateral and ipsilateral cortical tissues and subjected to ELISA for the measurement of TNF-α. * *p* < 0.05 vs. saline or the contralateral tissues of the vehicle group and # *p* < 0.05 vs. the ipsilateral tissues of the vehicle group, *n* = 6.

To further explore the outcomes of hyperglycemia on postischemic brain injury, insulin and glucose were predelivered to the rats prior to ischemia. Pretreatment with insulin alleviated—but glucose augmented—postischemic brain infarction (Figure 6A), caspase 3 activity (Figure 6B), and TNF-α protein (Figure 6C). These findings suggest that hyperglycemia augments postischemic apoptosis, inflammation, and brain injury while propranolol possesses ameliorative effects.

### 3.5. Cerebral Ischemia Impaired Insulin Action in Gastrocnemius

**Figure 7:**
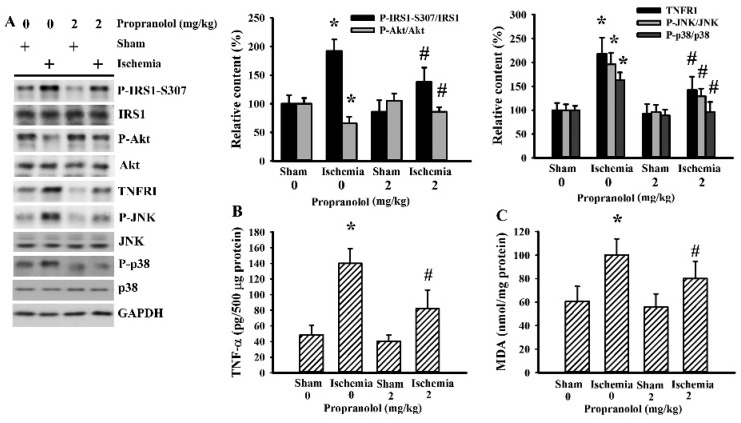
Propranolol alleviated postischemic gastrocnemius inflammation. Rats receiving a normal saline vehicle or a propranolol (2 mg/kg) intraperitoneal injection were subjected to sham and permanent cerebral ischemia for 24 h. (**A**) Proteins were extracted from the gastrocnemius muscles and subjected to Western blot with the indicated antibodies. (**B**) Proteins were extracted from the gastrocnemius muscles and subjected to ELISA for the measurement of TNF-α. (**C**) The contents of MDA in the gastrocnemius muscles were measured. * *p* < 0.05 vs. the sham/saline group and # *p* < 0.05 vs. the ischemia/saline group, *n* = 6.

Skeletal muscles are the main peripheral organs/tissues for the uptake and utility of glucose, and once impaired, results in hyperglycemia and insulin resistance [31]. Upon examining the gastrocnemius muscles, cerebral ischemia caused a reduction in Akt phosphorylation and an increase in IRS1 phosphorylation at 307 serine residue, JNK phosphorylation, p38 phosphorylation, and TNFRI. Propranolol reversed the altered protein content and protein phosphorylation in cerebral ischemic rats (Figure 7A). There was an elevated level of TNF-α (Figure 7B) and MDA (Figure 7C) in the gastrocnemius muscles of cerebral ischemic rats. The altered parameters in the postischemic gastrocnemius muscles were alleviated by propranolol. In conclusion, the impaired insulin signaling in the gastrocnemius muscles represented an alternative mechanism for the induction of postischemic hyperglycemia and insulin resistance, with propranolol improving the impairment.

### 3.6. Propranolol Decreased Isoproterenol-Induced Cytokine Production

**Figure 8:**
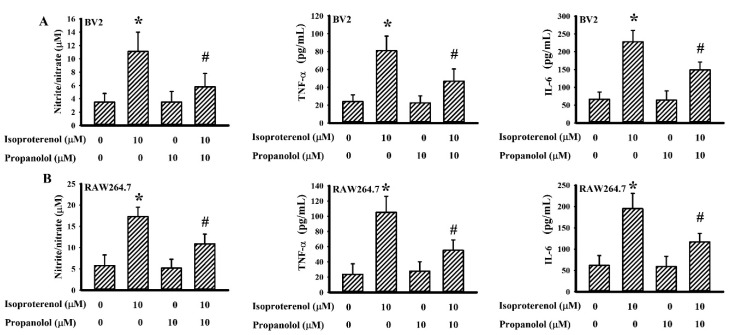
Propranolol alleviated cytokine production in BV2 and RAW264.7 cells. BV2 (**A**) and RAW264.7 (**B**) cells were pretreated with a vehicle or propranolol (10 μM) for 30 min before being incubated with isoproterenol (0 and 10 μM) for an additional 24 h. Supernatants were collected and subjected to a Griess reagent or ELISA for the measurement of nitric oxide (NO), TNF-α, and interleukin-6 (IL-6). * *p* < 0.05 vs. untreated control and # *p* < 0.05 vs. isoproterenol control, *n* = 4.

To directly evaluate the effects of propranolol on cytokine production, the murine BV2 microglial cell line along with the RAW264.7 macrophage cell line were stimulated with the adrenergic agonist isoproterenol. Isoproterenol treatment caused increased production of NO, TNF-α, and IL-6 in BV2 (Figure 8A) and RAW264.7 (Figure 8B) cells. Concurrently, propranolol alleviated the production of NO, TNF-α, and IL-6 in isoproterenol-stimulated BV2 (Figure 8A) and RAW264.7 (Figure 8B) cells. These findings indicate that adrenergic activation is able to induce cytokine production by macrophages/microglia, as well as show that there is an inhibitory effect of propranolol on isoproterenol-provoked cytokine production.

## 4. Discussion

Our groups have described a state of hyperglycemia and insulin resistance, along with elevated circulating levels of adrenaline and noradrenaline, in rat models of cerebral ischemia. The postischemic hyperglycemia and insulin resistance are closely linked with adipose inflammation, hepatic inflammation, and hepatic gluconeogenesis. Our study, along with other relevant studies, further indicate that pretreatment with the nonselective β-adrenoreceptor antagonist propranolol improves postischemic hyperglycemia, impaired glucose tolerance, and insulin resistance [14,15,16,22,28]. The studies presented here further extend earlier findings that propranolol possesses anti-inflammatory, neuroprotective, and hypoglycemic effects in vitro and in vivo. Using rat models experiencing cerebral ischemia, pretreatment with propranolol offered protection against brain infarction, edema, and apoptosis. The neuroprotection caused by propranolol was accompanied by a reduction in fasting glucose, fasting insulin, glucose tolerance impairment, plasma CRP, plasma free fatty acids, plasma corticosterone, brain oxidative stress, and brain inflammation. Pretreatment with insulin alleviated—while glucose augmented—postischemic brain injury and inflammation. Additionally, the impairment of insulin signaling in the gastrocnemius muscles was noted in rats with cerebral ischemia, as well as its improvement due to the use of propranolol. The anti-inflammatory effects of propranolol were further demonstrated in isoproterenol-stimulated BV2 and RAW264.7 cells through decreasing cytokine production. These findings provide new insight into the anti-inflammatory, neuroprotective, and hypoglycemic mechanisms of propranolol in combating neurodegenerative diseases, such as stroke.

It has been believed for a long time that hyperglycemia due to critical illness is an adaptive and protective response for patients who are combating stress. However, stress hyperglycemia transforms into a pathogenic factor with a high risk of mortality and morbidity after acute stroke. Tight glycemic control in the management of acute ischemic stroke remains controversial due to the hypoglycemic consequences [32]. Despite the debate, the control of stress hyperglycemia and having it return to the normal range is of benefit to ischemic stroke subjects, as seen in experimental studies [4,33,34]. The homeostatic regulation of circulating glucose levels is strictly counterbalanced by gluconeogenesis, glycogenolysis, and glucose uptake as it occurred in the liver, adipose tissues, skeletal muscles, and kidney. The liberation of free fatty acids and adipokines, along with oxidative stress and inflammation in the adipose tissues, interfere with insulin actions and glucose uptake. Hepatic oxidative stress and inflammation both facilitate hepatic glucose output as a result of their negative effects on insulin-inhibited gluconeogenesis and insulin-promoted glycogenesis. The hyperglycemic contribution of dysregulated adipose and liver tissues, along with the intervention by propranolol in cerebral ischemic rats, has been reported in our previous studies [15,16]. The skeletal muscles are the largest organs/tissues to fulfill the peripheral action of insulin, with an aim to uptake circulating glucose [31]. Akt plays a dominant role in the execution of insulin actions and is under the control of the insulin receptor and IRS1 phosphorylation cascade. Otherwise, TNF-α/TNFRI/JNK/p38 represents an alternative cascade to negatively impact Akt through the targeting of IRS1 [31,35,36,37]. The impaired insulin action in the gastrocnemius muscles was highlighted by the reduction of Akt phosphorylation after cerebral ischemia. The decreased Akt phosphorylation in the gastrocnemius muscles was accompanied by oxidative stress, elevated TNF-α/TNFRI/JNK/p38 signaling, and inhibitory IRS1 serine-307 hyperphosphorylation. Since inflammatory cytokines and oxidative stress contribute substantially to the impairment of insulin action [31,35,36,37], the positive effects of propranolol towards improving gastrocnemius insulin action and hyperglycemia could be attributed to its suppressive effects on cytokine production and oxidative stress.

Being a regulator of glucose metabolism, insulin also displays neurotrophic actions to help protect against neurodegenerative injury. Our previous study demonstrated a reduction of IRS1/Akt signaling in the brains of cerebral ischemic rats [14]. Thus, the neuroprotective effects of insulin against cerebral ischemia may be to boost the IRS1/Akt-mediated neurotrophic activity. On a parallel level, glucose injection exacerbated postischemic inflammation, apoptosis, and brain injury. A growing body of evidence has suggested that there is a proinflammatory effect of hyperglycemia, even in the CNS [2,5,6,7,8,9,10]. Here, the inhibition of brain TNF-α production implies that the neuroprotective effects of insulin against cerebral ischemic injury may be secondary to its hypoglycemic effect. However, its pleiotropic effects on postischemic alterations and brain injury warrant further investigation.

Cerebral ischemia in rats is closely linked with the development of peripheral and CNS inflammation, along with the activation of sympathetic tone, HPA axis, and stress hormones [14,15,16,28]. In remaining consistent with the studies, plasma levels of CRP and corticosterone, along with the brain and gastrocnemius content of TNF-α, were elevated in cerebral ischemic rats. This study further demonstrated that propranolol brought reduction. Although activation of the sympathetic nervous system has been described in stroke-associated spleen atrophy and immune suppression [17,18], there have also been many studies indicating the proinflammatory effect of adrenergic action. Activation of the β-adrenergic receptor promotes proinflammatory responses in the microglia, primes microglia to immune challenge, and induces neuroinflammation in vitro and in vivo [23,25,26,38]. Regarding stroke, augmented β2-adrenergic signaling increases stroke size, while β-adrenoreceptor antagonists provide neuroprotection against cerebral ischemia [19,20,21]. In this study, the anti-inflammatory consequences of propranolol in cerebral ischemic rats were evidenced through a reduction in inflammatory mediators in the brain, blood, and gastrocnemius muscles. Apart from macrophages/microglia, β-adrenoreceptor agonists also induce cytokine production by the skeletal muscles [39,40]. Therefore, through intraperitoneal administration, propranolol is able to traffick through the blood during circulation to reach the gastrocnemius muscles and the CNS, followed by abrogating cytokine production in macrophages/microglia, skeletal muscles, or yet to be identified cell types. Data taken from BV2 and RAW264.7 cell studies have revealed the proinflammatory potential of the β-adrenoreceptor agonist isoproterenol, along with the immune suppressive effect of propranolol against adrenergic activation. Independent from the β-adrenergic system, the anti-inflammatory effect of propranolol is observed in trauma, sepsis, and infection [41,42,43]. These phenomena suggest that the anti-inflammatory effect of propranolol is universal and offers the opportunity to act as an anti-inflammatory agent.

Macrophages/microglia can be categorized into two phenotypes: proinflammatory and anti-inflammatory. Neuroinflammation can arise from an imbalance between proinflammatory and anti-inflammatory phenotypes favoring the former, with a reversal in the balance ameliorating disease progression, including stroke [44]. The presence of IRF5, IRF8, P2X4R, P2X7R, and P2Y12R promotes microglia polarization towards proinflammatory phenotypes, while CD163, CD206, arginase 1, Ym-1, Nrf2, Sirt1, and Heme Oxygenase-1 (HO-1) shift microglia to anti-inflammatory phenotypes [27,45,46,47,48]. We found that cerebral ischemia-associated neuroinflammation was accompanied by the activation of microglia and astrocytes, along with the reduction of neurons and compromise of the blood–brain barrier tight junction. Propranolol reversed the microglia polarization switch involving suppression of IRF8 expression and promotion of Nrf2, Sirt1, and CD163 expression. Adrenaline enhances the response of macrophages under Lipopolysaccharide (LPS) stimulation and communicates with the toll-like receptors to establish proinflammatory phenotypes [49,50]. However, under endotoxemia and acute lung injury, the β2-adrenergic receptor favors the M2 regulatory macrophages [51]. The controversial effects of adrenergic systems on macrophages/microglia polarization, immune activation, and immune suppression complicate their specific roles in immunity. Although current findings suggest that the macrophages/microglia polarization switch is associated with a reduction in inflammatory responses, the detailed anti-inflammatory mechanisms of propranolol against cerebral ischemia still requires additional investigation.

Despite their potential benefits, stroke-associated hyperglycemia and inflammation are commonly linked with harmful consequences. Human study reveals a clinical benefit in patients taking β-blockers before stroke onset, resulting in improvement of poststroke hyperglycemia [52]. It has been reported that systemic adrenergic blockade before or after stroke normalizes extracellular ionic dynamics and facilitates recovery from acute ischemic stroke [53]. Additionally, renal ischemia/reperfusion injury also causes hyperglycemia along with elevated catecholamines [54]. The relevant studies highlight a role of adrenergic blockade against ischemic insults. Through this study, we have provided experimental evidence outlining the suppressive effects of propranolol on hyperglycemia, inflammation, and brain injury in a rat model experiencing cerebral ischemia. The neuroprotective capabilities of propranolol are closely linked with its actions on macrophages/microglia through its ability to switch polarization from proinflammatory towards anti-inflammatory phenotypes, while also reducing TNF-α production. The anti-inflammatory effects of propranolol were also duplicated in isoproterenol-stimulated microglia and macrophage cell lines. Propranolol improved postischemic hyperglycemia by subsiding oxidative stress and TNF-α-impaired insulin action in the gastrocnemius muscles. It should be noted that hemodynamic change is another target for the action of β-blockers. Our previous study found a negligible difference of blood pressure between sham-operated and ischemic stroke rats [16]. Therefore, the hemodynamic effect of propranolol in the current study appeared to be minor. However, the assumption should be concerning because of the use of only ischemic stroke rats. Its effects in hemorrhagic stroke rats and ischemia/reperfusion rats should be taken into consideration. Although there still remain limitations to our experiments, we believe the nonselective β-adrenoreceptor antagonist propranolol to be a proposed anti-inflammatory and neuroprotective candidate for the treatment of neuroinflammation-accompanied neurodegenerative diseases such as stroke. Before this theory can be translated into clinical practice, however, deeper investigative insight into its anti-inflammatory actions is still required.
